# A novel *Filobacterium* sp can cause chronic bronchitis in cats

**DOI:** 10.1371/journal.pone.0251968

**Published:** 2021-06-09

**Authors:** Martina Načeradská, Sona Pekova, Patrizia Danesi, Tommaso Furlanello, Roberta Calleo, Patricia Martin, Fumio Ike, Richard Malik

**Affiliations:** 1 Department of Veterinary Sciences, Faculty of Agrobiology, Food and Natural Resources, Czech University of Life Sciences in Prague, Prague, Czech Republic; 2 Veterinary Clinic of MVDr. Martina Načeradská, Prague, Czech Republic; 3 Tilia Laboratories, Laboratory for Molecular Diagnostics, Pchery, Czech Republic; 4 Istituto Zooprofilattico Sperimentale delle Venezie, Legnaro (PD), Italy; 5 San Marco Veterinary Clinic and Laboratory, Veggiano, Italy; 6 Veterinary Pathology Diagnostic Services, Sydney School of Veterinary Science, University of Sydney, Sydney, NSW, Australia; 7 Experimental Animal Division, RIKEN BioResource Research Center, Tsukuba, Japan; 8 Centre for Veterinary Education, University of Sydney, Sydney, NSW, Australia; University of Bari, ITALY

## Abstract

**Background:**

Cilia-associated respiratory bacillus (CARB; now known as *Filobacterium rodentium* gen. nov., sp. nov.) is a primary pathogen of rodents. A CARB-like organism was reported in post-mortem lung samples of cats using light and electron microscopy. Here we explore by molecular procedures if a *Filobacterium* sp. is a part of the normal feline lower respiratory microbiome and whether it could in some cats contribute to the development of chronic bronchial disease.

**Methodology:**

A *Filobacterium* sp. was identified in three Czech cats clinically diagnosed as having chronic neutrophilic bronchitis. Bronchoalveolar lavage fluid (BALF) specimens obtained from these cats were subjected to panbacterial 16S rDNA PCR followed by Sanger sequencing of the V5 to V8 region. After these cats were treated with specific antimicrobials, their clinical signs resolved promptly, without recurrence. Next, BALF specimens from 13 Australian and 11 Italian cats with lower respiratory disease and an additional 16 lung samples of Italian cats who died of various causes were examined using next generation sequencing (NGS). Subsequently, a *Filobacterium*-specific qPCR assay was developed and used to re-test BALF specimens from the 11 Italian cats and lung tissue homogenates from the additional 16 deceased cats.

**Principal findings:**

An amplicon of 548 bp with 91.24% sequence agreement with *Filobacterium rodentium* was obtained from all three patients, suggesting the novel *Filobacterium* sp. was the cause of their lower respiratory disease. The novel *Filobacterium* sp., which we propose to call *F*. *felis*, was detected in 3/3 Czech cats with chronic neutrophilic bronchitis, 13/13 Australian cats and 6/11 Italian cats with chronic lower respiratory disease, and 14/16 necropsy lung specimens from Italian cats. NGS and qPCR results all showed identical sequences. The *Filobacterium* sp. was sometimes the preponderant bacterial species in BALF specimens from cats with lower airway disease. There was an association between the presence of large numbers (greater than 10^5^ organisms/mL) of *Filobacterium* and the presence of neutrophilic and/or histiocytic inflammation, although only a subset of inflammatory BALF specimens had *F*. *felis* as the preponderant organism.

**Conclusion:**

The novel *Filobacterium* sp. comprises a finite part of the normal feline lower respiratory microbiome. Under certain circumstances it can increase in absolute and relative abundance and give rise to neutrophilic and/or histiocytic bronchitis, bronchiolitis and bronchopneumonia. These findings strongly suggest that *F*. *felis* could be an underdiagnosed cause of feline bronchial disease.

## Introduction

Cilia-Associated Respiratory bacillus (CARB) is a historic term referring to a morphologically similar group of bacteria that colonise the ciliated respiratory epithelium of many animal species. It is the cause of chronic bronchopulmonary disease in naturally and experimentally infected rats where its role as a primary pathogen is well established [1–4]. The organism that causes disease in the rats has been studied systematically and a recent publication [5] gave it the more specific name *Filobacterium rodentium* gen. nov., sp. nov., a member of *Filobacteriaceae* fam. nov. within the phylum *Bacteroidetes*.

*Filobacterium rodentium* is a fastidious, Gram-negative, filamentous organism that is motile without flagella, via a gliding motion [1]. It was first reported in 1980, but similar organisms had actually been visualized in electron micrographs published in the 1960s and even earlier [6]. There have been attempts to cultivate the agent on conditioned Vero cells media [5] and other artificial substrates [7], but with inconsistent results. Sometimes we use the original CARB nomenclature to be consistent with the extensive older literature, especially when mentioning related species found in other animal species.

As well as rats [1–4], rabbits [8] and mice [2, 3, 9], CARB has been found in other mammals including, goats [10, 11], cattle [12, 13], pigs [13–15] and deer [16]. In cattle and calves, infection is associated with tracheitis [12, 13], while in goats and kids there is an association with pneumonia [10, 11]. Experimental infections have been established in rats [17–19], goats [20], mice, rabbits [21, 22], guinea pigs [21], hamsters [22] and gerbils [23]. *Filobacterium rodentium* causes bronchopneumonia in wild rats in various parts of the world [2, 24, 25] and it contributes to the morbidity and mortality associated with polymicrobial respiratory infections in wild and laboratory rodents [26].

A single case of CARB-associated respiratory disease has been reported in a cat that died during anaesthesia for dentistry. At necropsy, light and electron microscopy (EM) examinations revealed bronchitis and bronchiolitis associated with organisms morphologically consistent with CARB amongst the cilia of the lower airways [27]. Material was not available for culture, while serology and molecular studies were not undertaken. In comparison with CARB documented in other animal species, the filamentous bacteria in this cat were smaller in diameter. Because the authors were able to identify CARB-like organisms morphologically in the cat that died, but also in the lung of some healthy cats, they concluded, conservatively, that the significance of these organisms in the pulmonary lesions could not be determined with certainty[27].

*F*. *rodentium* is commonly associated with chronic respiratory disease (CRD) in rats, often together with *Mycoplasma pulmonis* infection [17, 28]. Polymicrobial infections caused by CARB-like organisms in association with mycoplasmas and/or viruses [28] are recognised in other animal species [2, 28]. Both CARB and mycoplasmas affect the function of ciliated respiratory epithelium, resulting in disturbed mucociliary clearance, predominantly neutrophilic inflammation, and the development of symptomatic CRD. Infection may also be subclinical, with minimal or even absent macroscopic changes. Chronic suppurative bronchitis and bronchiolitis accompanied by peri-bronchial cuffing with lymphocytes and plasma cells are observed histologically when disease is present [4, 28]. The presence of CARB in the lower airways can impair lung function by stimulating the production of proinflammatory cytokines [29]. Antibody-mediated immunity appears non-protective against subclinical or clinical infection due to *F*. *rodentium* and indeed the detection of specific antibodies is used as a diagnostic test in laboratory rodents [30].

The primary route of transmission of CARB appears to be direct contact with infected animals or contaminated fomites, while airborne transmission is considered of lesser importance [21]. In laboratory animals, commercial antibody tests are routinely used for diagnosis [30, 31], together with animal species specific real-time quantitative polymerase chain reaction (qPCR) testing [3, 32]. Silver staining, EM [4] or immunofluorescent assay (IFA) techniques using histological preparations of lung tissue [31] can also be used for diagnosis.

According to a recent review, approximately 1 to 5% of the feline population is affected by chronic lower respiratory tract disease [33]. Indeed, even higher numbers of cats might be affected as respiratory distress and coughing can be misidentified as ‘a hairball problem’ by owners, so the actual prevalence of airway disease is probably under-estimated [34]. Feline bronchial disease can be further classified into four overlapping categories–(i) feline asthma, characterised by eosinophilic airway inflammation associated with increased mucus, bronchospasm, coughing and distal air trapping, (ii) chronic bronchitis, where neutrophils are the predominant cell type within airways and coughing is the main clinical sign [33–36], (iii) parasitic bronchitis, and (iv) secondary bacterial bronchitis [37]. A key distinguishing feature of asthma is reversible airflow limitation due to functional bronchoconstriction, thought to result from a type I hypersensitivity reaction in airway walls associated with mast cell degranulation [33, 34, 38].

Both forms of feline bronchial disease can be complicated by secondary infection with bacteria including mycoplasmas; indeed, there is some speculation that mycoplasmas might trigger feline asthma in some instances [39–41]. Studies of the microbiome of lower and upper respiratory tract of cats [42, 43], dogs [44], healthy people [39, 40] and human asthma patients [41] have been conducted, but to date, CARB or *Filobacterium* spp. have not been described in any peer-reviewed studies, except for a conference Abstract by the first two authors [45] and a poster presentation at the University of Missouri Veterinary Research Scholars program (http://vrsp.missouri.edu/wp-content/uploads/2019/11/Reid.pdf).

Our study started with an index group of three cats with chronic bronchial disease. Using molecular microbiology methods applied to deep unguided bronchoalveolar fluid (BALF) samples, a novel *Filobacterium* sp. was identified as the principal causative agent in these cats. After successful targeted treatment of the patients, we extended investigations to larger cohorts of animals with chronic lower airway disease. Our aim was to (*i*) investigate the frequency of *Filobacterium* reads by quantitative pan-bacterial next generation sequencing (NGS) in cats with and without CRD, (*ii*) develop a novel *Filobacterium*-specific quantitative real-time PCR (qPCR) as a reliable tool to detect the presence of this potential pathogen in diagnostic specimens and *(iii)* briefly explore the possibility of etiological significance of this organism in feline respiratory disease.

## Materials and methods

### Ethical approval

In this study, no fluid or tissue specimens were obtained specifically with the intention to progress this project. Three owned cats underwent standard procedure for diagnosing cough in the practice of first author (MN). Residual BALF samples were obtained from veterinary laboratories after routine diagnostic testing had been complete. Rather than discarding such samples as is routinely done, they were frozen and archived and eventually used in this study. Lung samples from cats that had died from various causes and subjected to routine post-mortem examination were obtained, frozen and archived. Thus, all the samples used had been collected for routine veterinary investigations, and the material obtained would otherwise have been discarded. In our various jurisdictions, such samples do not require animal ethics approval for utilisation in research when collected from companion animals if the owner’s details have been de-identified.

### Informed consent

This work did not involve the use of animals, but material was only obtained from animals being investigated for clinical reasons and for their benefit. Therefore, informed consent was not required. No animals or humans are identifiable within this publication, and therefore additional informed consent for publication was not required.

### Outline of clinical Investigation

Our study started with three cats with chronic bronchitis. Molecular microbiology methods were applied to deep unguided BALF samples. The study was subsequently extended to a larger cohort of animals, using archived BALF samples from 13 Australian and 11 Italian cats with bronchial disease, together with 16 opportunistic feline lung samples originating from cats which died of vehicular trauma and other causes. BALF specimens were subjected to quantitative pan-bacterial NGS assay and *Filobacterium*-specific qPCR analyses.

#### *Filobacterium*-positive cats from Czech Republic

Three cats representing the index cases comprised an 18-month-old neutered domestic short hair (DSH) (Cat 1), a 3-year-old neutered DSH (Cat 2) and a 6.5-years-old neutered Bengal (Cat 3). All cats were domiciled indoors as single pets, with no history of travelling outside the Czech Republic and no access to outdoors. All three had signs of chronic bronchial disease including intermittent cough. Cats were investigated by chest radiography, haematological and serum biochemistry testing and rapid NT-proBNP point-of-care testing (Cat 3; IDEXX Laboratories, Westbrook, Maine, USA). The latter is a test which detects elevations in brain natriuretic peptide (NT-proBNP) caused by atrial stretch associated with cardiac disease. The goal of all testing was to exclude heart failure as a potential cause of coughing [46]. To exclude the possibility of *Aleurostrongylus abstrusus* and other lungworm infections, Baermann testing of fresh faecal samples for larvae (Cat 1 and 2) or empiric anthelmintic therapy (Cat 3; emodepside and praziquantel; Profender Spot-on, Bayer) were undertaken.

Unguided BALF [47] during general anaesthesia was collected from all cats for microbiological culture, cytological examination, and molecular investigations. Specifically, a commercial multiplex qPCR (FelinePlex II, Tilia Laboratories, Czech Republic) for a panel of feline respiratory pathogens (*Mycoplasma spp*., *Chlamydia spp*., feline herpesvirus (FHV)-1, feline calicivirus (FCV) and *Bordetella bronchiseptica*) was carried out. Additionally, panbacterial PCR amplification and sequence analysis targeting the V5 to V8 region of the 16S rDNA gene was undertaken.

#### Expanded testing of respiratory specimens from cats with lower respiratory disease or without respiratory signs

Respiratory samples were collected opportunistically from various sources. None of these specimens were collected specifically to progress this investigation. Samples from the lower respiratory tract were obtained from three different groups of cats. Firstly, BALF samples were made available from 13 Australian cats with bronchial disease investigated at several centres in Sydney and submitted for cytological examination and conventional bacterial culture. Some of these had been obtained using a bronchoscope, while others had been obtained using an unguided catheter technique. In both instances, sampling occurred via an instrument inserted through the endotracheal tube. Secondly, BALF specimens were obtained via bronchoscopy from 11 Italian cats with chronic bronchial disease investigated at the Veterinary Clinic San Marco, Italy. Thirdly, lung specimens (2 x 0.5 by 0.5 cm sections, weighing 0.2 to 0.5 g taken from the right lung of each cat) were obtained at necropsy at the Diagnostic Department of IZSVe, Italy from 16 cats that died because of cancer, acute largely non-respiratory viral disease, chronic kidney disease or following vehicular trauma.

At least 1 mL of BALF was available from each patient. BALF and lung tissue samples were preserved at −20°C prior to DNA extraction. The signalment and clinical findings for these various cohorts are given in Tables 1–3.

**Table 1:** BAL samples of 13 cats with bronchial disease from Australia.

No.	Breed	Age (y)	Sex	History and Clinical signs	WBCs	% Neutrophils	%Macro-phages	%Eosinophils	Culture	*F*. *felis* qPCR Copies/mL of starting sample	% NGS reads panB
**1**	Oriental	9	FN	Chronic rhinitis no longer responsive to doxy and dexamethasone, chronic airway disease responsive to fluticasone/salmeterol,	Moderate to numerous	52	23	9	NG	6.2 x 10^6^	99%
**2**	Burmese	8	MN	Coughing for 6 months	Moderate	50	34	9	Heavy mixed bacterial growth—mainly smelly, yellow Gram (-)	5.8 x 10^5^	75%
**3**	Maine Coon	4/12	MN	Chronic tachypnoea, normal X-rays and CT, mild inflammation on bronchoscope	Low to Moderate	15–30	56–69	6–7	Light mixed bacterial growth	1.3 x 10^7^	98%
**4**	Burmese	10	MN	Chronic, coughing and sneezing, upper and lower respiratory tract noises/disease, prominent bronchial tree, mucus above palate. 2 years later got a *Pseudomonas* brain abscess by extension from frontal sinus.	Moderate	34	39	24	Light growth *Pseudomonas*	7.5 x 10^5^	86%
**5**	Burmese	16	MN	Chronic cough, haemoptysis, diffuse bronchointerstitial/alveolar opacities, fibrous tissue in bronchus, bulla?	Moderate to numerous	30	55	scattered	Light to moderate mixed bacterial growth incl. *Pasteurella* types	3.6 x 10^6^	78%
**6**	Persian	1	MN	Chronic URT, LRT disease, mucus in nasal cavity, trachea, bronchi, bronchointerstitial pattern, R middle lobe consolidation, treated with multiple antibiotics	Numerous	96	4		Light mixed growth incl Group G strep., oxidase +ve Gram (-) rod, *Penicillium* etc.	5.2 x 10^6^	96%
**7**	DMH	N/a	FN	Chronic cough, suspect asthma, treated with fluticasone and doxycycline (stopped), diffuse pulmonary pattern	Moderate to numerous	1	88	10	Moderate mixed bacterial growth	4.6 x 10^5^	20%
**8**	Bengal	4	MN	Episodes of coughing and noisy breathing, increased RR, partial response to doxy, bronchial pattern	N/a	low numbers	mainly	scattered	No growth	2.8 x 10^4^	8%
**9**	DSH	1	FN	Chronic cough, CT suggests asthma/bronchitis, mucopurulent exudate in bronchus	Numerous	95	5		Possibly tiny colonies in the deposit of the anaerobic plate but ‘disappeared’ with time	2.0 x 10^5^	5%
**10**	DMH	3/12	M	Chronic LRT disease, moist cough, small volume pleural effusion, bronchointerstitial pattern, no response to metronidazole, Clavulox, doxycycline	Moderate to numerous	40	49	4	Light mixed bacterial growth incl *E*. *coli* and *Pasteurella*. *Aleurostrongylus* and *Eucoleus aerophilus*	2.2 x 10^5^	4%
**11**	Selkirk Rex	7	MN	Cough, dyspnoea, increased lung sounds, severe consolidation esp. R caudal lung, intraluminal mineralisation, fluid, and yellow plaques in bronchi PM: pulmonary adenocarcinoma and granulomatous pneumonia	Low to moderate	18	57	17	Light mixed growth including α-*Strep*., *Bacillus* and *Aspergillus fumigatus*	1.3 x 10^4^	8%
**12**	DSH	13	FN	Chronic airway disease, no response to doxy and prednisolone	Numerous	54–82	2–6	0–9	Moderate growth, *Pasteurella* types	3.4 x 10^3^	4%
**13**	Ragdoll	5	MN	Harsh lung sounds, increased RR, coughing lethargy, bronchial pattern	Low to moderate	1	93	2	Heavy mixed growth including *Enterobacter* and *Klebsiella oxytoca*	3.4 x 10^4^	7%

Blue shading indicates cats where *F*. *felis* was the preponderant bacteria present; DSH domestic short air; DMH domestic medium hair; MN male neuter; M intact male; FN female neuter; F intact female.

**Table 2:** BAL samples of 11 cats with bronchial disease from northern Italy.

No.	Breed	Age (y)	Sex	History and Clinical signs	WBCs	% Neutrophils	% Macrophages	% Eosinophils	Culture	*F*. *felis* Copies/mL of starting sample	% NGS reads panB
**1**	Main Coon	12	MN	Progressive weight loss for few months, Breathing difficult and chronic cough. No responsive to enrofloxacin.	Numerous	80	20	0	*Pasteurella multocida*	6.9 x 10^6^	58%
**2**	Main Coon	8	MN	Progressive weight loss, depression and sialorrhea for two days. Chronic dyspnoea, laryngeal mass with purulent exudation.	Low to moderate	15	80	4	-ve	1.9 x 10^5^	31%
**3**	European Shorthair	5	MN	Chronic dyspnoea and dyspnoea after simple play activities.	Moderate	5	90	5	*Acinetobacter baumannii* complex	5.9 x 10^4^	82%
**4**	European Shorthair	1	MN	Chronic cough, first dry, then moist. No responsive to short course of cortisone. Partially responsive to cefovecin.	Moderate	30	30	40	-ve	9.3 x 10^2^	1%
**5**	European Shorthair	15	FN	Chronic cough, steroids responsive. Endoscopy shows exudative bronchitis and bronchial collapse.	Moderate to numerous	80	15	0	*Pasteurella multocida*	5.9 x 10^4^	1.3%
**6**	European Shorthair	12	FN	Acute cough, fever and anorexia. No response to cephalexin. TC scan revealed lung tumor.	Numerous	85	10	0	*Serratia marcescens*	9.3 x 10^2^	2.6%
**7**	European Shorthair	13	FN	Chronic snuffler and nasal discharge: lymphoplasmacytic and neutrophilic rhinitis.	Low	70	5	20	*Stenotrophomonas maltophila*	Nil	0
**8**	European Shorthair	2	MN	Chronic cough, partially responsive to glucocorticoids at high doses and doxycycline.	Moderate	1	50	45	-ve	Nil	0
**9**	European Shorthair	4	FN	Progressive chronic cough, despite steroid aerosol therapy.	Moderate to Numerous	60	2	30	-ve	Nil	0
**10**	European Shorthair	1	FN	Chronic paroxysm and coughing, on progressive worsening. No antibiotics responsive.	Numerous	70	20	0	-ve	Nil	0
**11**	British Shorthair	7	MN	For about a month has experienced respiratory noises, lately coughing. No responsive to steroid injections.	Low	50–60	30	0	-ve	Nil	0

Blue shading indicates cats where *F*. *felis* was the preponderant bacteria present or present at a concentration greater than 10^5^/mL; DSH domestic short air; DMH domestic medium hair; MN male neuter; M intact male; FN female neuter; F intact female.

**Table 3:** Lung samples of cats from Italy.

No.	Family/Street cat	Breed	Age	Sex	Neutered	Nutrition status	Lung—Macroscopic	Lung—Histology	Bacteriology (culture) and parasites	Cause of death/suspicious	*F*. *felis* qPCR copies/mL of starting sample	%NGS reads
**1**	Street cat	European short hair	Adult	MN	Yes	Good	Pulmonary congestion	nd	nd	Adenocarcinoma (Liver)	6.4 x 10^5^	65%
**2**	Street cat	European short hair	Adult (10 years)	M		Poor	Pulmonary congestion	Partial destruction of the parenchyma with interstitial infiltration of lymphoid cells	nd	FeLV-associated thoracic lymphoma	6.4 x 10^5^	N/a
**3**	Family	European short hair	Young (< 1 year)	M		Good	Pneumonia and emphysema	Lymphocytic Interstitial pneumonia and fibrinopurulent thrombo-embolic dissemination	Lung: *Staphylococcus* spp.	Lymphocytic Interstitial pneumonia	1.9 x 10^5^	39%
**4**	Street cat	European long hair	Young adult (12–24 months)	F-pregnant	no	Good	Congestion of parenchyma.	Congestion of parenchyma. Alveolar oedema foci.	nd	Enteritis	5.9 x 10^4^	2.6%
**5**	Street cat	European long hair	Adult	F	not reported	Good	Congestion of parenchyma	Alveolar oedema.	nd	Vehicular trauma	3.3 x 10^4^	1.9%
**6**	Street cat	European short hair	Adult	F	no	Good	Pulmonary congestion and oedema	Oedema and alveolar congestion	nd	Necrotic superficial enteritis. Nephritis.	1.8 x 10^4^	3.5%
**7**	Street cat	European short hair	Young (< 1 year)	F	no	Good	Pleural and pericardial serum-hematic effusion; pulmonary congestion.	nd	**Lung:** *Staphylococcus spp*. **Parasites in gut:** *Toxocara cati* and *Taenia taeniaeformis*	Enteritis	5.5 x 10^3^	N/a
**8**	Street cat	European short hair	Adult	F	not reported	Poor	Pulmonary congestion and oedema	nd	nd	Chronic nephritis	1.7 x 10^3^	3.8%
**9**	Street cat	European short hair	Young (< 1 years)	M	no	Good	Pulmonary congestion	nd	**Gut**: *Taenia taeniaeformis* and *Toxocara cati*	enteritis	1.7 x 10^3^	0.27%
**10**	Street cat	European short hair	Young (< 1 years)	M	no	Poor	Not reported	Autolysis. Presence of numerous bacteria	nd	Catarrhal haemorrhagic gastritis and enteritis	9.3 x 10^2^	2%
**11**	Street cat	European short hair	Young (< 1 year)	F	no	Good	Abdominal haemorrhage and hemoperitoneum	nd	nd	Vehicular trauma	9.3 x 10^2^	N/a
**12**	Family	European short hair	Adult	MN	yes	Good	Not reported	Alveolar oedema.	nd	Hepatitis and catarrhal haemorrhagic enteritis	2.8 x 10^2^	3.5%
**13**	Family	European short hair	Adult	M	no	Good	Pulmonary congestion and haemorrhage at apical lobes	Diffuse haemorrhage	**Lung:** Escherichia coli, Enterococcus. **Spleen:** Enterococcus	Vehicular trauma	1.6 x 10^2^	0.02%
**14**	Family	European short hair	Young (< 1 year)	F	no	Good	Bilateral pleural effusion	Alveolar atelectasis, presence of bacteria in the alveoli	**Lung:** *Escherichia coli*; Enterococcus. **Gut:** *Clostridium perfringens*	Necrotic enteritis	1.6 x 10^2^	N/a
**15**	Family	Maine Coon	Young (< 1 years)	F	no	Good	Inflamed areas with pulmonary consolidation in both lungs. Fibrin exudation. Foam and whitish material in bronchial lumen.	Focal granulomatous pneumonia with fibrinous exudation, abundant bacterial and congestive pneumonia. Rare fibrin thrombus in blood vessels	**Lung:** Corynebacterium spp. and Enterobacter spp. **Spleen:** *Escherichia col*i and *Enterobacter kobei*.	Gastro-enteritis	Nil	0
**16**	Family	Scottish Fold	Young (< 1 years)	M	no	Poor	Oedema and pulmonary congestion at apical lobes	Atelectasis. Emphysema and alveolar oedema.	**Lung:** -ve. **Brain**: -ve. **Liver:** -ve. **Gut**: S*taphylococcus* spp., *Streptococcus canis*	Peritonitis	Nil	0

### Molecular protocols

#### DNA extraction and panbacterial PCR

DNA was isolated from 0.5–1.0 ml of BALF or approx. 150 mg of lung tissue using a Qiagen DNA mini kit (Qiagen, DE) and tissue homogenisers, according to the manufacturer’s recommendations. Pandetection of bacteria using polymerase chain reaction (PCR) followed by direct Sanger sequencing of V5 to V8 region of the 16S rDNA gene was carried out after DNA extraction, as described previously [48]. The PCR amplicons obtained were directly sequenced on an ABI 3500 genetic analyser (Applied Biosystems, USA) and aligned to the NCBI-based microbiota database (https://www.ncbi.nlm.nih.gov/).

#### Phylogenetic data analysis

Phylogenetic data analysis was described in detail [5]. Briefly, alignment of sequence data was performed using Genetyx-Mac program (Genetyx Corp., Tokyo, Japan). The phylogenetic data were constructed by using the neighbour-joining method followed by bootstrap resampling to estimate the confidence of tree topologies.

#### Pan-bacterial (panB) NGS

Respiratory microbiomes in all feline BALF (24 in total) and lung tissues (16 in total) were investigated using NGS. PanB products covering the V5-V8 portion of bacterial 16S rDNA were produced as described earlier for Sanger sequencing. PCR products of approximately 650 bp were purified using QIAquick Gel Extraction Kit (Qiagen, DE) and subjected to NGS library building using NEBNext® Fast DNA Library Prep Set for Ion Torrent kit (NEB, USA) according to the manufacturer’s instructions. Final libraries were quantified using Ion Plus Fragment Library kit (Thermo Fisher Scientific, USA) and 10 pM library pool was used as a template for emulsion PCR using Ion PGM™ Hi-Q™ View OT2 Kit (Thermo Fisher Scientific, USA). After bead enrichment (OT2 instrument, Thermo Fisher Scientific, USA), a NGS sequencing chip was loaded, typically v316 or v318 (Thermo Fisher Scientific, USA), to reach the sequencing dynamic range of 4–5 orders of magnitude. NGS sequencing was performed using the Ion Torrent PGM platform (Thermo Fisher Scientific, USA) using the Ion PGM™ Hi-Q™ View Sequencing Kit chemistry (Thermo Fisher Scientific, USA). The raw data (pair-ended) obtained were end- and quality-trimmed.

#### Contig building

From the quality filtered NGS reads, only reads 200 bp and longer (up to 650 bp) were used for the read overlap (plus and minus strands) and contig building using CodonCode Aligner (CodonCode Corporation, USA). Individual contigs were directly aligned to the bacterial reference sequences depository located on the NCBI server (https://www.ncbi.nlm.nih.gov/) and downloaded locally to produce Excel-compatible data sets.

#### Log re-quantitation of the NGS data

NGS data is only proportional, showing the percentage of individual reads in the whole analysed sample. Thus, to create quantitative log data, calculated as the copy number of a given microorganism in 1 mL of starting biological material of the various microbes found in the sample (corresponding to classical quantitative microbiology cultivation read-outs), at least one microorganism was selected for use as a target for quantitative qPCR. The qPCR log quantity was then used as an internal calibrator for a given sample to mathematically re-quantify the remaining microbes found in the sample. As the laboratory had developed a significant portfolio of pathogen-specific quantitative qPCR assays over the years, a specific calibrator qPCR assay was readily selected for each sample.

#### Design and development of quantitative qPCR assays for the novel *Filobacterium* sp

This assay was performed on feline BALF specimens (11 Italian cats with lower airway disease) and necropsy lung tissue homogenates (16 Italian cats). To select the optimal target region of the microbe to be detected using a quantitative qPCR approach, pathogen-unique sequences were identified using multiple alignment of all reference sequences deposited at NCBI for CARB and *F*. *rodentium* using CodonCode Aligner. Regions with the intra-species homology, but inter-species heterology, were selected as the targets for the design of pathogen-specific primers and pathogen-specific TaqMan hybridization probes. For the construction of calibration curves, synthetic standards containing primers- and probe-recognition sequences and flanking heterogeneous sequences were designed and purchased from Eurofins Genomics, DE. Synthetic standards were serially diluted from 10^6^ copies to 10^3^ copies per PCR reaction and calibration curves for the individual targets were constructed. The calibration curve equations were then used for recalculation of C_*T*_ values measured in the authentic samples to the number of copies of the target sequence (for each microorganism) in 1 mL of starting biological material (taking into consideration the initial sample dilution and DNA elution volume). The sensitivity of the quantitative qPCR assay developed was measured as the lowest concentration of the quantitation standard that would yield an amplification signal. Typically, the detection limit of a qPCR assay used in the study ranged between 5–15 copies of target sequence in each PCR reaction. The variability of the individual qPCR assays was tested using two different biologically relevant concentrations of the quantitation standard, typically 10^6^ and 10^3^ copies, carried out in triplicate. An inter-sample maximum variability of 0.3 cycle was considered acceptable. To test the specificity of a pathogen-specific qPCR assay, all positive signals (15 cases) were re-sequenced using Sanger sequencing, employing one of the primers used for the quantitative qPCR. Only assays with 100% specificity were approved for further diagnostic use.

The oligonucleotides designed and positively validated for the CARB-specific qPCR assay are:

CARB-specific forward primer: CAGCACCTTATTAGCAGTATGTTTCC
CARB-specific reverse primer: CCGCACAAGAGGTGGAACATGTGG
CARB-specific probe: FAM—TCAGAGGCGATCTACTAACATTCTAGCCTAGGTA—BHQ1

These primers differ from those utilized in previous studies of CARB in other animal species, and PCR target regions of 16S rDNA are different [3, 32]

The PCR conditions were as following: initial denaturation at 94°C for 5 min, followed by 45 cycles with the profile: 94°C 20 sec, 56°C 30 sec, 72°C 30 sec, with fluorescence acquisition at 56°C. The PCR chemistry used was based on the thermostable FastStart™ Taq DNA Polymerase (Sigma, DE), with MgCl_2_ at a final concentration of 2.5 mM.

## Results

### 1. Clinical course for *Filobacterium*-positive Czech cats

Three cats had clinical signs of chronic bronchial disease with intermittent cough. Chest radiographs demonstrated a prominent bronchial pattern consistent with bronchitis or asthma. Haematology and serum biochemistry were unremarkable (S1 and S2 Tables). Cats 1 and 2 had normal echocardiographic examinations, while Cat 3 had a negative NT-proBNP SNAP test result, suggesting none of the cats had cardiac disease. Faecal samples of Cats 1 and 2 were negative for lungworm larvae using the Baermann apparatus, while Cat 3 failed to improve after treatment with emodepside and praziquantel (Profender® SPOT-ON, Bayer).

Cytology of unguided BALF specimens in all three cats demonstrated neutrophilic bronchitis, with macrophages comprising the remaining 10% of cells present. All BALF specimens were negative on routine bacterial culture. Multiplex qPCR testing of BALF samples for common feline bacterial and viral respiratory pathogens was negative.

**Fig 1:**
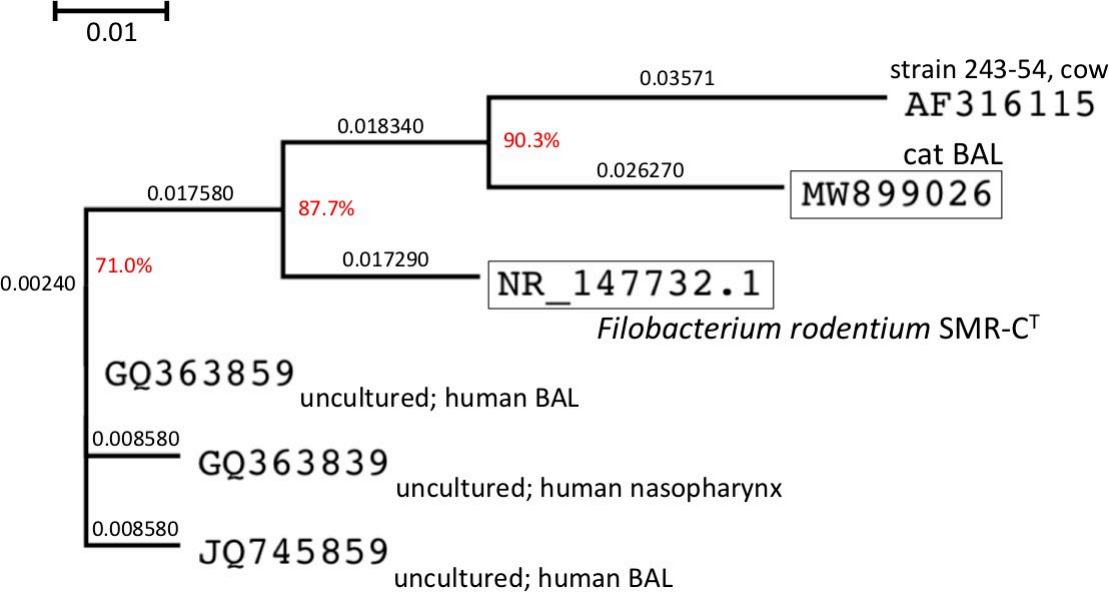
Neighbour-joining phylogenetic tree based on 16S rRNA gene sequences showing the relationships of the organism derived from cats and other *Filobacterium* spp. Bootstrap values are displayed in red, based on 1,000 replicates. Bar, 0.01 substitutions per nucleotide position. MW899026 (representative 16S rDNA sequence data of the organisms derived from the three cats) is alligned using ClustalW program with *Filobacterium rodentium* SMR-C^T^ (type strain and type genus) 16S rRNA gene reference sequence (NR_147732.1) and sequences of putative *Filobacterium* spp. listed in SILVA SSU r138.1. The aligned data were trimmed by manual inspection (nucleotide position 774 to 891 of NR_147732.1).

Pan-bacterial 16S rDNA PCR followed by Sanger sequencing identified a novel *Filobacterium* sp. as the only bacterial species present in all three BALF samples, suggesting it as a potential causative pathogen. The amplicon size and sequence were identical for all three feline patients. The PCR amplicon had 548 base pairs (GenBank accession number MW899026; S1 Fig). This MW899026 sequence was compared with known sequences using a BLASTn search. The nearest type strain was *Filobacterium rodentium* (sequence agreement 91.24%), while the nearest culturable bacterial strain was strain 243–54 isolated from cow (94.16% sequence agreement) estimated as a *Filobacterium* sp. according to SILVA SSU r138.1. These findings show the organism found in all three BAL samples was a novel *Filobacterium* sp. we have tentatively named *Filobacterium felis*. The phylogenetic relations between this *Filobacterium* sp., *F*. *rodentium* and the other closely related CARB from a cow are illustrated in the dendrogram in Fig 1.

Cats 1 and 2 were treated with a course of trimethoprim/sulfamethoxazole (TMS; 15 mg/kg combined, twice daily for 6 weeks (trimethoprim 20 mg, sulfamethoxazole 100 mg per tablet as a fixed dose combination; Biseptol, Polfa) according to a protocol developed for rodents and rabbits [8, 49–51]. There was complete resolution of clinical signs within three weeks. Due to poor compliance in Cat 3, therapy was switched to spiramycin (75,000 IU/kg) and metronidazole (12.5mg/kg) orally once a day ([fixed dose combination]; Stomorgyl, Merial), in combination with cefovecin (8 mg/kg subcutaneously every 14 days for 3 doses; Convenia, Zoetis) according to recommendations for a similar group of bacteria [52]. There was a corresponding improvement in the chest radiographs of the three cats during and after therapy.

At the time of writing (April 2021), Cats 1–3 have remained free of all clinical signs following the completion of antimicrobial therapy for follow-up periods 20, 19 and 21 months, respectively.

### 2. NGS analysis of BALF and lung samples and *Filobacterium* sp.- specific qPCR testing

To determine if the results in the three index cases could be generalised to a larger cohort of cats with lower respiratory signs, we determined the range of bacterial respiratory species present in feline BALF samples using quantitative NGS pandetection of bacteria (panB) with species assignment based on V5-V8 16S rDNA sequencing. Firstly, we tested 13 archived feline BALF samples from Australian cats. All 13 cats had been investigated for lower respiratory disease by radiology and collection of bronchoscopic or deep unguided BALF specimens with subsequent cytological evaluation and aerobic and anaerobic bacterial culture.

**Fig 2:**
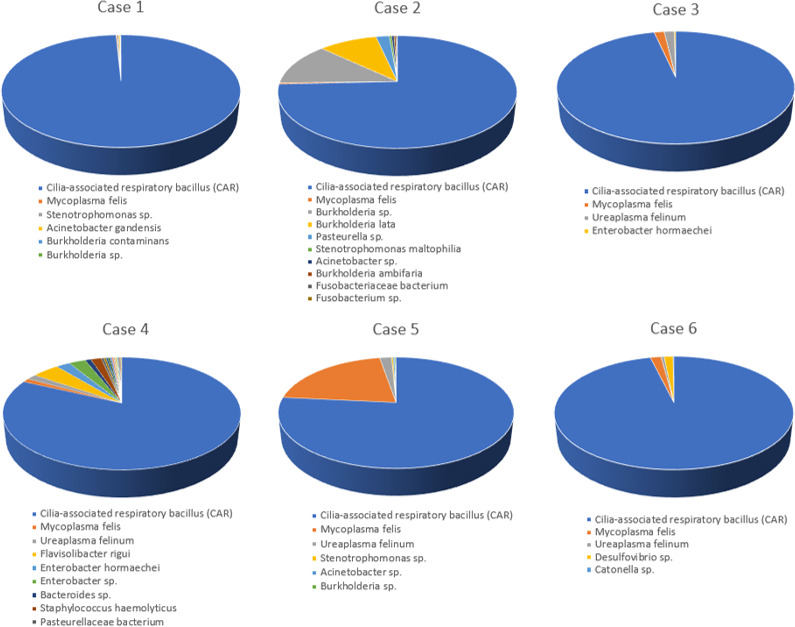
Pie charts of the percentage reads of various constituent 16S rDNA sequences in NGS analysis of feline BALF specimens from cats with lower airway disease and where *Filobacterium felis* was the preponderant organism. For consisteny, *F*. *felis* reads are shown in navy blue, *Mycoplasma felis* reads are shown in orange and *Ureaoplasma felinum* reads are shown in grey. For simplicity, bacteria with reads less than 0.2% were not illustrated.

**Fig 3:**
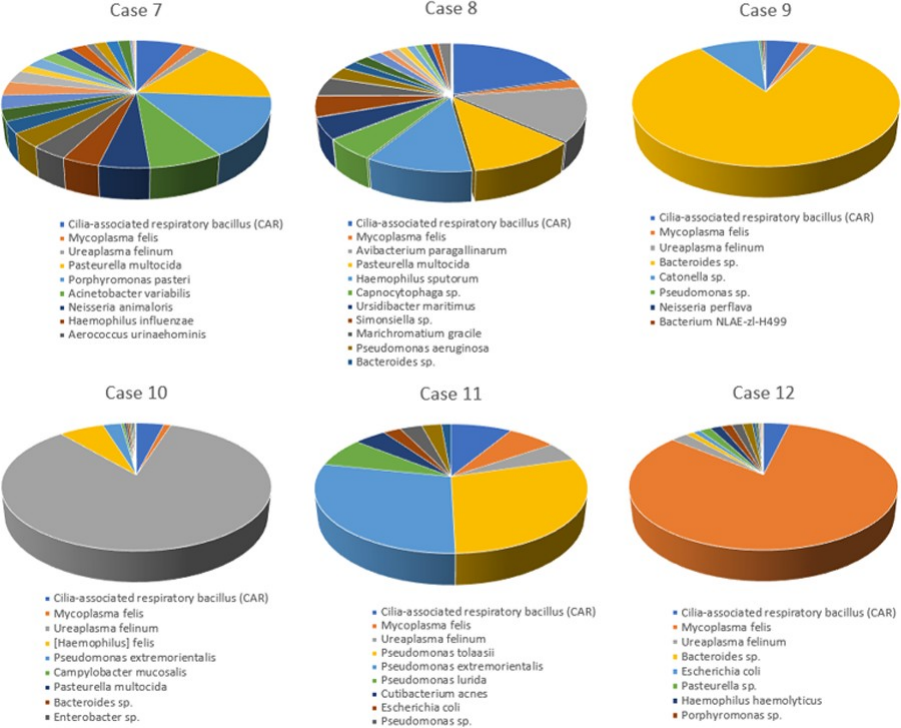
Pie charts of the percentage reads of various constituent 16S rDNA sequences in NGS analysis of feline BALF specimens from cats with lower airway disease and where *F*. *felis* was *not* the preponderant organism. Legend same as for Fig 2.

NGS data (https://dataview.ncbi.nlm.nih.gov/object/PRJNA721302) demonstrated that the novel *Filobacterium* sp. was present in 13/13 of BALF specimens, often as the preponderant organism (Table 1 and Fig 2). Indeed, *F*. *felis* made up more than 75% of the NGS reads in 6/13 samples (75 to 99% of NGS reads; Fig 2 and Table 1). In the remaining seven samples, *F*. *felis* was present in lower numbers (4 to 20% of NGS reads) and usually accompanied by mycoplasmas, ureaplasmas, *Pasteurella multocida* and various obligate anaerobic bacterial species (Table 1 and Fig 3).

The *F*. *felis* was detected 6/11 (55%) BALF samples from Italian cats with lower respiratory disease, and in two it was the preponderant organism (comprising 82% and 58% of NGS reads). The difference between *F*. *felis* prevalence in Australia *versus* Italy was significant (13/13 *v* 6/11; p = 0.01; 2-tailed Fisher’s Exact test) (Table 2).

Subsequently, a *F*. *felis*-specific quantitative qPCR assay was developed. This assay could be applied more readily and less expensively to BALF specimens, including the 11 cats from Northern Italy with lower respiratory signs (Table 2), and lung specimens obtained opportunistically at necropsy from 16 cats from Northern Italy that had died of various mostly non-respiratory conditions (Table 3). The necropsy lung samples from cats were selected as a ‘control group’ of cats that had not shown prominent lower respiratory signs. Where samples were tested with both NGS and qPCR (n = 21), there was excellent agreement between the two methods (k = 0.999). Where both qPCR and NSG data was available, the qPCR estimation of copies per mL of starting sample were tabulated for analysis, except for BALF samples from Australian cats where only NGS data were available, as qPCR had not been undertaken. All qPCR positive samples were confirmed to have identical sequences (MW899026) by Sanger sequencing.

Considering the combined cohort of 24 cats with lower respiratory disease with BALF specimens available, the calculated copy number of *F*. *felis* was > 10^5^ copies/mL of BALF in 11/24 cats (46%). The highest quantity detected was 1.3 x 10^7^ copies/mL; in the remainder, copy number ranged from approximately 10^3^ to 10^5^ in eight cats (8/24; 33%), while in 5/24 (21%) *F*. *felis* was not detected in the BALF specimens.

Of the 16 Italian cat lung specimens collected at necropsy, *F*. *felis* was detected in 14/16 (88%) samples using species-specific qPCR. *F*. *felis* was detected at a concentration of >10^5^ copies/mL in two lung samples (cats 1 and 3, Table 3) and in an additional lung specimen from a FeLV-positive cat with thoracic lymphoma (cat 2, Table 3). In 11 necropsy lung specimens, the calculated copy number for *F*. *felis* ranged from 10^2^ to 10^4^ copies/mL of initial specimen, while two specimens were *Filobacterium*-negative. In terms of the percentage of NGS reads, *F*. *felis* made up 65% and 39% of NGS reads in two specimens, including an adult cat with hepatic adenocarcinoma and a young cat with lymphocytic interstitial pneumonia, respectively (Table 3). NGS *F*. *felis* reads ranged from 0.02 to 3.8% in eight other cats and data were unavailable for four specimens.

**Fig 4:**
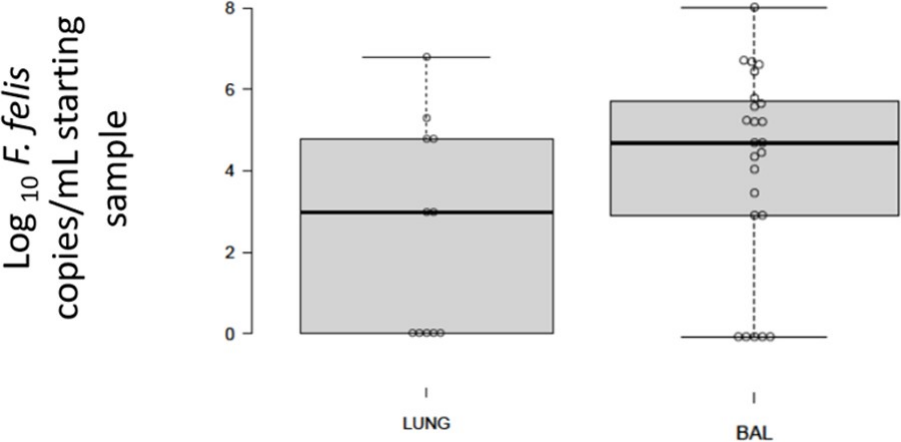
*F*. *felis* copy number as detected using NGS or qPCR in BALF samples. A comparison of log_10_ *F*.*felis* copies as detected using NGS or qPCR in BALF samples from 24 cats with lower airway disease (Australia and Northern Italy) compared to 16 necropsy lung specimens from cats in northern Italy. Note that as a generality, cats with lower airway disease tended to have substantially higher copy numbers of *F*. *felis* than present in necropsy lung specimens.

**Fig 5:**
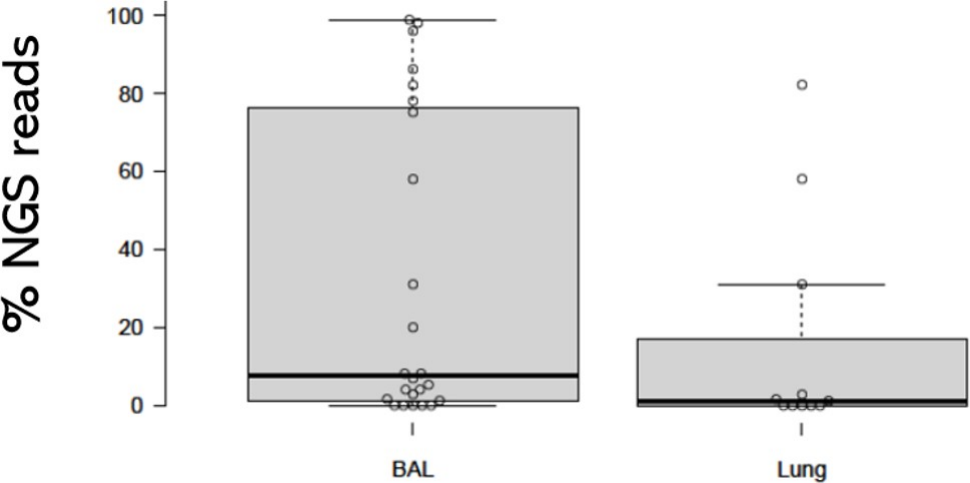
The percentage of NGS reads attributable to *F*. *felis* in BALF specimens. The percentage of NGS reads attributable to *F*. *felis* in BALF specimens from 24 cats with lower airway disease comapred to in 16 lung necropsy specimens. It was not uncommon for *F*. *felis* to be the preponderant organism in cats with lower respiratory disease, but this was rarely the case in lung specimens from the control necropsy cat, except for the cat that died of intersitial pneumonia.

The number of *F*. *felis* copies detected using NGS or qPCR in 24 feline BALF specimens from cats with lower airway disease were compared with those from the 16 necropsy lung specimens (Fig 4). Cats with lower airway disease had median copy numbers of *F*. *felis* in BALF approximately two-orders of magnitude more than those present in necropsy lung specimens from ‘control’ cats. According to the *F*. *felis* NGS data, it was not uncommon for this organism to be the preponderant bacterium in cats with symptomatic lower respiratory disease, but this was unusual in lung specimens from necropsy cats, except for the cat that died with interstitial pneumonia (Fig 5). These results suggest *F*. *felis* is a commensal of the lungs of many normal cats, being present in concentrations of less than 10^4^ copies per gram of lung tissue in most instances, where it presumably colonises the lining of the airways. Under certain circumstances, however, it can behave as a primary or opportunistic pathogen and give rise to neutrophilic bronchitis, bronchiolitis, and possibly interstitial pneumonia.

## Discussion

This work started from an initial observation that three Czech cats with neutrophilic bronchitis had a preponderance of *Filobacterium felis* nucleic acid in BALF specimens [45]. The diagnosis of this novel bacterial species in association with feline disease had never been made previously (except in a single cat at necropsy), as the organism is non-cultivatable on routine synthetic laboratory media and is too thin to be readily detected in cytological preparations stained with Gram or with Romanowsky dyes such as DiffQuik, Wrights or Giemsa and examined using conventional light microscopy. The collaboration between the primary author, a veterinarian (MN), and a human molecular microbiologist (SP) co-located geographically was serendipitous. This circumstance enabled the molecular detection of this novel pathogen, initially by panbacterial 16S rDNA PCR and sequence analysis. Although a lack of NGS data for these three cats may represent a limitation of this study, the results obtained certainly indicates the potential role of *F*. *felis* as a primary respiratory pathogen in cats. Further support for this contention is provided by the finding that the three cats were successfully treated using antimicrobial therapy directed against *Filobacterium* sp., with permanent resolution of clinical signs with long term follow-up.

To further investigate the potential association between the novel *Filobacterium* sp. and feline disease, we extended the collaboration to Australian and Italian colleagues with archived and curated collections of BALF specimens from cats with chronic non-allergic lower respiratory disease signs, and lung tissue specimens collected opportunistically from cats at necropsy. Using NGS initially, and subsequently a specially designed species-specific qPCR for *F*. *felis*, we demonstrated that this organism was present in small numbers as part of the normal lower respiratory microbiome of normal cats but represented the preponderant organism in a subset of cats with chronic bronchitis. The presence of CARB in normal cat lung had been shown morphologically in bronchi and bronchioles: 2/9 normal lungs, 1/7 lungs with bronchitis and bronchiolitis, and 1/2 lungs with pneumonia had CARB as revealed by light microscopy [27].

Accordingly, we posit that *F*. *felis*, a novel feline-associated *Filobacterium* sp. is an unrecognised cause of chronic bronchitis in cats, and this should be explored prospectively in future studies, using appropriate molecular tools, bronchoscopy, ultrastructural analysis of pelleted BALF specimens and computed tomography of the chest. As a corollary, TMS might be worth reassessing as an empiric fixed-dose drug combination for cats with neutrophilic bronchitis, using suitable formulations with a sugar or gelatine coating to mask the bitter taste. During the gestation of this manuscript, one of the authors has consulted on three feline patients with chronic neutrophilic bronchitis where TMS therapy proved to be unequivocally successful, but in which molecular diagnostics were not undertaken (RM, unpublished observations).

How could previous studies of normal cats and cats with chronic lower airway disease have failed to detect *F*. *felis*, when related organisms have been well explored as a cause of respiratory disease in laboratory rodents and several other domestic species? There are several reasons why this pathogen might have been missed. Firstly, *Filobacterium* spp. cannot be cultured aerobically, anaerobically or microaerophilically *in vitro* using synthetic media. Secondly, the bacilli are so thin as to be virtually invisible using routine staining methods and conventional light microscopy in cytological and histological tissue specimens. Thirdly, serological methods that detect antibodies against *Filobacterium* spp. have not been applied to cats even though they are used routinely in laboratory rodents. Fourthly, no research group we know of routinely subjected samples from cats with chronic bronchitis to electron microscopy. Finally, peer-reviewed studies of the feline respiratory microbiome have up until now failed to detect *Filobacterium* spp. using the methodologies adopted in normal or diseased cats. Together, these represent critical omissions which have hindered our ability to recognise this potential pathogen in feline patients.

For NGS microbiome analysis, Illumina V4 16S rDNA fragment sequencing has gained wide acceptance as the analytic mainstay [53]. The short-read length (maximum 200 bp) of this technology might, however, limit precision and extent of pan-microbial detection. The quantitative NGS microbiome approach used in our study leverages Ion Torrent PGM technology, which allows sequencing of a much longer V5 to V8 16S rDNA fragment (approx. 650 bp). This provides one possible explanation of why *F*. *felis* has not been detected in previous studies of the normal respiratory microbiome, or the microbiome of cats with lower airway disease [42, 43]. A conference Abstract from 2019, however, using Illumina technology to amplify the V4 region of 16S rRNA did indeed detect *Filobacterium* sp. sequences in normal lung and the lungs of cats with feline asthma (http://vrsp.missouri.edu/wp-content/uploads/2019/11/Reid.pdf). It is possible that the SILVA database r132 (Dec 13, 2017 version) used for operational taxonomic unit (OTU) assignment in earlier studies did not contain *Filobacterium* data, whereas more modern iterations (e.g., SSU r138.1) incorporate *Filobacterium* sequences.

The present study demonstrated *F*. *felis* was the preponderant pathogen in BALF specimens from three Czech cats with neutrophilic bronchitis and a larger number of similar archived samples from cats with chronic bronchitis with a mixed inflammatory pattern (neutrophils and macrophages) from Australia and Italy. This provides strong circumstantial evidence that *F*. *felis* is the cause of chronic bronchitis in at least some of these cats. The improvement in the three index cats with targeted antimicrobial therapy provides further support for this contention. The failure of the remainder of cats to develop progressive disease despite the presence of *F*. *felis* suggests this bacteria often behaves as a low-grade pathogen, and even administration of inhaled or systemic corticosteroids does not usually lead to acute exacerbation of *Filobacterium* disease (RM, unpublished observations).

The current results infer that the difference in the clinical status of an individual patient might be reflection of the relative and absolute quantity of *Filobacterium* found in target tissues, with low numbers being a feature of asymptomatic colonisation and large numbers a feature of symptomatic primary or secondary infection. Thus, in our study cohorts, higher quantities of *F*. *felis* (≥ 10^5^ copies/mL sample) were most often observed in a subset of patients with chronic lower respiratory signs associated with inflammation characterised by neutrophils and/or macrophages. In contrast, much lower numbers were present in the lungs of normal cats without respiratory disease (usually ≤ 10^4^ copies of *F*. *felis*/mL sample). Quantitative real-time PCR is therefore crucial for the investigation of patients with chronic bronchitis and our suggestion is that primers for *F*. *felis* be added to commercial multiplexed diagnostic respiratory PCR panels for cats.

The behaviour of *F*. *felis* in normal cats and cats with chronic bronchitis has parallels with two well characterised ‘stealth pathogens’. Sticking with the respiratory system, the fungal pathogen *Pneumocystis* is present in low numbers in the lungs of normal dogs, cats and people, presumably after maternal colonisation soon after birth. But in the setting of severe immunodeficiency, either nutritional, drug-induced or due to genetic defects in immunity, trophic forms of *Pneumocystis* multiply unchecked and give rise to life-threatening pneumonia [54]. Considering the gastrointestinal tract, *Helicobacter* species can be present in superficial mucus overlying the villi of the stomach of cats, dogs and humans, giving rise to limited or absent inflammation under most circumstances, but occasionally can be associated with the development of gastritis, gastric ulceration and eventually gastric lymphoma in certain individuals [55].

No previous study has reported the antemortem diagnosis of *Filobacterium-*associated bronchitis in cats, nor its successful treatment with targeted antimicrobial therapy. To date, only one case of infection in a cat by a CARB has been published, the diagnosis having been made post-mortem [27]. Interestingly, the CARB seen in EM preparations from this case were morphologically distinct from *F*. *rodentium* found in rats, which might accord with the large sequence mismatch (8.8% over 548 bp) between our *F*. *felis* sequence and that of *F*. *rodentium* [27]. In the three samples from the Czech cats, all *F*. *felis* amplicons had an identical sequence, and the same was true for all Italian and Australian cases subjected to NGS and qPCR testing.

*Filobacterium*/CARB is well established as a cause for lower respiratory tract signs in guinea pigs, gerbils, mice and rabbits [21]. Mice-origin CARB is distinct and does not cause disease in rats [28]. Rabbit CARB similarly appears to be host specific and does not give rise to disease in rats [32]. DNA analysis of rabbit and rat isolates revealed slight differences in their sequences [32]. The species-specific differences in strains of CARB/*Filobacterium* spp. isolated from different host species is reminiscent of the situation for the fungal commensal organism *Pneumocystis*, where species-specific reductive evolution has resulted in adaptions to different mammalian hosts (i.e. convergent evolution) [54]. Similar phylogenetic parallels exist for the spiral organisms which live amongst the cilia in the gastric mucus of different mammalian hosts [55].

The most common presenting complaints for cats with lower respiratory tract disease are dyspnoea, coughing and a fast and/or laboured breathing pattern. Disease conditions that must be excluded by diagnostic testing include pulmonary oedema, verminous pneumonia due to lung and tracheal worms, pleural space disease such as pyothorax, heartworm disease and neoplasia of the lungs or mediastinum [33]. Auscultation, chest radiography, computed tomography, ultrasonography, bronchoscopy, cytological analysis of BALF specimens after staining with DiffQuik and Gram stains, followed by culture for bacteria and fungi and multiplex qPCR testing for feline respiratory pathogens (including viruses), and allergy testing (serological and intradermal skin testing) are the diagnostic techniques commonly used in clinical practice. Respiratory function testing is not readily available to most practitioners and is difficult to apply to cats [56]. Strictly speaking, BALF is obtained using an endoscope wedged in a bronchus, although more commonly it is collected in practice by an unguided sterile plastic or red rubber catheter wedged deep into the airways or even by lavage via the endotracheal tube. The combination of imaging, BALF analysis, cytology and culture usually distinguishes asthma from chronic bronchitis [47, 57]. Multiplex PCR testing can detect viral pathogens and bacteria which are difficult to grow on routine media, such a mycoplasmas and ureaplasmas [58]. The BALF cytology of the three Czech cats and some individuals of the Australian and Italian cohorts where *F*. *felis* was the preponderant organism showed variable combination of neutrophils, macrophages and even eosinophils in BALF specimens, consistent with suppurative bronchitis. None of the BALF specimens had eosinophils as the preponderant inflammatory cell, so any association between *F*. *felis*, eosinophilic inflammation and ‘feline asthma’ cannot be determined based on data to hand. The poster from the Missouri group would seem to indicate *Filobacterium* sp. was not the preponderant bacteria present in cats with feline asthma.

In laboratory rodents, it has been found that infection with CARB in many respects resembles mycoplasma bronchopneumonia, causing similar clinical signs and histopathological changes [2]. In rats [2, 28], mice [28] and pigs [13], CARB is often present in association with *Mycoplasma* spp in individuals with lower respiratory disease. We made similar observations in the Australian cohort of cats with lower airway disease, where *F*. *felis*, was often preponderant but accompanied by mycoplasmas or ureaplasmas (6 cases), or where mycoplasmas or ureaplasmas were preponderant but accompanied by *F*. *felis* (2 cases). Studies of feline inflammatory airway disease have shown that mycoplasmas are impossible to see convincingly using conventional light microscopy and are present normally in low numbers as part of the upper respiratory microbiome. Mycoplasmas are typically associated with neutrophilic inflammation of the airways and lung parenchyma but are difficult to grow on routine unenriched media and often die during transit to the laboratory [42, 59–61]. The co-occurrence of *F*. *felis* and mycoplasmas is a challenge therapeutically as sulpha drugs are considered the drug of choice for treating *Filobacterium*/CARB in rabbits and rodents but have no or limited efficacy for mycoplasmas, so to treat both pathogens combination therapy using TMS and either azithromycin, doxycycline or a fluoroquinolone is required. Such combination therapy is not always well tolerated, and it may therefore be prudent to use a sequential approach. A further problem can be the absence of a suitable coated TMS formulation in some jurisdictions, as TMS has the potential to cause profuse salivation in cats unless the tablet is coated with sugar or gelatine. In the absence of suitable formulations, or perhaps in any case, we recommend TMS be administered to cats in a gelatine capsule lubricated with butter, margarine, or olive oil, or alternately to be given by daily subcutaneous injection. The value of nebulisation therapy [62] and coupage in cats infected with *F*. *felis* also needs to be investigated, for such techniques are an important adjunct to therapy when treating mycoplasmas which also live in mucus lining the ciliated respiratory epithelium [63].

In rats and rabbits, co-infection with viruses (Sendai virus) and CARB is observed [28]. Therefore, concurrent involvement with viruses such as FHV-1 or calicivirus should be considered possible in cats with acute *Filobacterium* disease [64, 65] and this should be explored in future research. *Bordetella bronchiseptica* is another well-recognised causative agent of pneumonia in cats [66], and PCR or routine bacterial culture should be a standard part of a laboratory diagnostic workflow for BALF samples to determine its presence and relative abundance.

In ruminants and wildlife, the precise role of CARB is unknown [12, 16]. In contrast, in mice, rats, rabbits, Guinea pigs, hamsters and gerbils [21–23, 28] the role of *Filobacterium*/CARB as a causative agent of chronic bronchopneumonia has been confirmed unequivocally. In laboratory animals, commercial testing laboratories (Charles River Laboratories and IDEXX) are routinely used for diagnosing CARB, but no commercial serology test has been developed for cats. Development of a species-specific point-of-care antibody test would be helpful for feline clinicians. In the absence of such tests, we elected to utilise panB 16S rDNA sequencing, NGS and subsequently species-specific qPCR testing. In other species it has been shown that CARB can be cultured on Vero E6 cells at 37°C in CO_2_/air (5:95, *v/v*), in embryonated eggs or on specially formulated artificial media [5].

The pathophysiology of *F*. *felis*-associated chronic bronchitis has not yet been elucidated. Our necropsy data suggests that a substantial proportion of normal cats harbour *F*. *felis* in low numbers as part of their lower airway microbiome [37].

As touched upon earlier, there are conceptual similarities between *Helicobacter* spp. in the stomach, *Pneumocystis* in the lungs and *F*. *felis* in the lower airways, with all these organisms being considered commensals in many normal feline individuals, but capable of causing inflammation and disease under certain circumstances. The pathophysiology of *Filobacterium*-associated disease has parallels with *Mycoplasma* infection of the respiratory tract [67] and *Helicobacter* infection of the stomach [68]. As commensals, these bacteria are commonly found in low numbers in healthy animals as part of the normal microbiome, causing little or no inflammation or clinical signs under routine circumstances. But *Filobacterium*/CARB, epitheliotropic mycoplasmas and spiral shaped bacteria might all be considered as ‘stealth pathogens’ of mucosal surfaces, living innocuously in mucus overlying ciliated epithelium until a change in the host parasite relationship permits expansion of their numbers, resulting in an inflammatory host response, tissue injury and emergence of clinical signs.

While the present results provide compelling evidence that *F*. *felis* is an infectious agent present in a subset of cats with chronic suppurative bronchitis, it is not possible to make any inferences on the importance of *F*. *felis* in cats with florid eosinophilic bronchitis (feline asthma), as our cohort of cats was largely devoid of such cases due to selection bias in obtaining BALF from cats thought *not* to have primary allergic airway disease [35, 36]. Although it was logical to concentrate on such cases to search for cryptic feline pathogens, the omission of classical feline asthma BALF specimens means we can make no inference on the extent to which *F*. *felis* may complicate or otherwise contribute to feline asthma.

The importance of *F*. *felis* in cats with upper respiratory disease and specifically the chronic post-viral snuffler entity requires reappraisal using appropriate NGS platforms and databases, as previous studies may have been incapable of detecting this organism. It therefore might have been missed in recent NGS studies of cats with sinonasal disease [42, 43].

We strongly advocate for the use of quantitative *F*. *felis*-specific qPCR and, where affordable, a quantitative NGS panbacterial approach in the investigation of feline respiratory tract disease, as cultivation-based microbiology does not offer definitive results in many instances, because of its inability to detect *F*. *felis*, mycoplasmas, and other fastidious organisms which can contribute to dysbiosis in the respiratory microbiome.

## Conclusions

A novel feline *Filobacterium* sp., which we tentatively call *F*. *felis*, can often be detected in the lower respiratory tract of cats (36/43 samples tested). This organism was the preponderant bacterial species in some but not all cases of chronic suppurative bronchitis. Using a quantitative pan-bacterial NGS approach complemented by targeted quantitative *F*. *felis*-specific qPCR assay, we were able to establish that this bacterium was often the principal infectious agent in BALF samples from a subset of cats with chronic bronchitis. *Filobacterium felis* would therefore appear to be a hitherto underdiagnosed potential cause of infectious lower airway disease in cats. It seems likely that some of these cases have been treated suboptimally with corticosteroid and bronchodilator therapy in the past, rather than targeted antimicrobial therapy directed against *F*. *felis* using trimethoprim-sulphonamide combinations or other effective antimicrobials.

In this work we have identified a novel *Filobacterium* sp as a potential causative agent of some cases of chronic bronchitis in cats. Although further analyses of *F*. *felis* including sequencing of the entire 16S rRNA gene, isolation and cultivation of the strain *in vitro*, ultrastructural studies, biochemical characterization, fatty acid composition analysis, and ultimately whole genome sequencing are required to fully characterise this organism. We hope to complete these studies in the future. As indicated in Fig 1, a human type *Filobacterium* sp. can also be detected in some human respiratory specimens. Studies of *Filobacterium* spp. in different species, including the cat, may provide fresh insights into the pathophysiology of disease produced by these fastidious host-adapted potential pathogens.

## Supporting information

S1 Fig MW899026, representative nucleotide sequence of the V5 to V8 region of the 16S rRNA gene amplicons obtained from Cats 1, 2 and 3, aligned with those of *Filobacterium rodentium* and a *Filobacterium* spp. (TIF)

S1 Table Biochemistry results of 3 Czech BAL examined cats. (DOCX)

S2 Table Haematology results of 3 Czech BAL examined cats. (DOCX)

## References

[1] Ganaway JR, Spencer TH, Moore TD, Allen AM. Isolation, propagation, and characterization of a newly recognized pathogen, cilia-associated respiratory bacillus of rats, an etiological agent of chronic respiratory disease. Infect Immun. 1985;47(2):472–9. doi: 10.1128/IAI.47.2.472-479.1985 ; PubMed Central PMCID: PMC263194. 3881350 PMC263194

[2] MacKenzie WF, Magill LS, Hulse M. A filamentous bacterium associated with respiratory disease in wild rats. Vet Pathol. 1981;18(6):836–9. doi: 10.1177/030098588101800616 . 7292900

[3] Goto K, Nozu R, Takakura A, Matsushita S, Itoh T. Detection of cilia-associated respiratory bacillus in experimentally and naturally infected mice and rats by the polymerase chain reaction. Exp Anim. 1995;44(4):333–6. doi: 10.1538/expanim.44.333 . 8575549

[4] Itoh T, Kohyama K, Takakura A, Takenouchi T, Kagiyama N. Naturally occurring CAR bacillus infection in a laboratory rat colony and epizootiological observations. Jikken Dobutsu. 1987;36(4):387–93. doi: 10.1538/expanim1978.36.4_387 . 3436371

[5] Ike F, Sakamoto M, Ohkuma M, Kajita A, Matsushita S, Kokubo T. *Filobacterium rodentium* gen. nov., sp. nov., a member of Filobacteriaceae fam. nov. within the phylum Bacteroidetes; includes a microaerobic filamentous bacterium isolated from specimens from diseased rodent respiratory tracts. Int J Syst Evol Microbiol. 2016;66(1):150–7. Epub 2015/10/16. doi: 10.1099/ijsem.0.000685 . 26476525

[6] Schoeb TR. Respiratory diseases of rodents. Veterinary Clinics of North America: Exotic Animal Practice.; 2000. p. 481–96. 10.1016/S1094-9194(17)30083-X PMC7110835 11228890

[7] Schoeb TR, Dybvig K, Davidson MK, Davis JK. Cultivation of cilia-associated respiratory bacillus in artificial medium and determination of the 16S rRNA gene sequence. J Clin Microbiol. 1993;31(10):2751–7. doi: 10.1128/JCM.31.10.2751-2757.1993 ; PubMed Central PMCID: PMC266006. 7504686 PMC266006

[8] Caniatti M, Crippa L, Giusti M, Mattiello S, Grilli G, Orsenigo R, et al. Cilia-associated respiratory (CAR) bacillus infection in conventionally reared rabbits. Zentralbl Veterinarmed B. 1998;45(6):363–71. doi: 10.1111/j.1439-0450.1998.tb00805.x . 9719769

[9] Griffith JW, White WJ, Danneman PJ, Lang CM. Cilia-associated respiratory (CAR) bacillus infection of obese mice. Vet Pathol. 1988;25(1):72–6. doi: 10.1177/030098588802500110 . 2830699

[10] Orós J, Fernández A, Rodríguez JL, Franklin CL, Matsushita S, Poveda JB. Association of cilia-associated respiratory (CAR) bacillus with natural chronic tracheitis in goats. J Comp Pathol. 1997;117(3):289–94. doi: 10.1016/s0021-9975(97)80025-4 . 9447491

[11] Orós J, Fernández A, Rodríguez JL, Rodríguez F, Poveda JB. Bacteria associated with enzootic pneumonia in goats. Zentralbl Veterinarmed B. 1997;44(2):99–104. doi: 10.1111/j.1439-0450.1997.tb00955.x . 9151536

[12] Hastie AT, Evans LP, Allen AM. Two types of bacteria adherent to bovine respiratory tract ciliated epithelium. Vet Pathol. 1993;30(1):12–9. doi: 10.1177/030098589303000102 . 8442323

[13] Nietfeld JC, Fickbohm BL, Rogers DG, Franklin CL, Riley LK. Isolation of cilia-associated respiratory (CAR) bacillus from pigs and calves and experimental infection of gnotobiotic pigs and rodents. J Vet Diagn Invest. 1999;11(3):252–8. doi: 10.1177/104063879901100308 . 10353357

[14] Hafner S, Latimer K. Cilia-associated respiratory bacillus infection and pneumonia in a pig. J Vet Diagn Invest. 1998;10(4):373–5. doi: 10.1177/104063879801000414 . 9786530

[15] Nietfeld JC, Franklin CL, Riley LK, Zeman DH, Groff BT. Colonization of the tracheal epithelium of pigs by filamentous bacteria resembling cilia-associated respiratory bacillus. J Vet Diagn Invest. 1995;7(3):338–42. doi: 10.1177/104063879500700307 . 7578448

[16] Bergottini R, Mattiello S, Crippa L, Scanziani E. Cilia-associated respiratory (CAR) bacillus infection in adult red deer, chamois, and roe deer. J Wildl Dis. 2005;41(2):459–62. doi: 10.7589/0090-3558-41.2.459 . 16107685

[17] Schoeb TR, Dybvig K, Keisling KF, Davidson MK, Davis JK. Detection of *Mycoplasma pulmonis* in cilia-associated respiratory bacillus isolates and in respiratory tracts of rats by nested PCR. J Clin Microbiol. 1997;35(7):1667–70. doi: 10.1128/JCM.35.7.1667-1670.1997 ; PubMed Central PMCID: PMC229818. 9196170 PMC229818

[18] Schoeb TR, Davidson MK, Davis JK. Pathogenicity of cilia-associated respiratory (CAR) bacillus isolates for F344, LEW, and SD rats. Vet Pathol. 1997;34(4):263–70. doi: 10.1177/030098589703400401 . 9240834

[19] Matsushita S, Joshima H. Pathology of rats intranasally inoculated with the cilia-associated respiratory bacillus. Lab Anim. 1989;23(2):89–95. doi: 10.1258/002367789780863600 . 2523502

[20] Fernández A, Orós J, Rodríguez JL, King J, Poveda JB. Morphological evidence of a filamentous cilia-associated respiratory (CAR) bacillus in goats. Vet Pathol. 1996;33(4):445–7. doi: 10.1177/030098589603300415 . 8817847

[21] Matsushita S, Joshima H, Matsumoto T, Fukutsu K. Transmission experiments of cilia-associated respiratory bacillus in mice, rabbits and guineapigs. Lab Anim. 1989;23(2):96–102. doi: 10.1258/002367789780863664 . 2523503

[22] Shoji-Darkye Y, Itoh T, Kagiyama N. Pathogenesis of CAR bacillus in rabbits, guinea pigs, Syrian hamsters, and mice. Lab Anim Sci. 1991;41(6):567–71. . 1667199

[23] St Clair MB, Besch-Williford CL, Riley LK, Hook RR, Franklin CL. Experimentally induced infection of gerbils with cilia-associated respiratory bacillus. Lab Anim Sci. 1999;49(4):421–3. . 10480649

[24] Brogden KA, Cutlip RC, Lehmkuhl HD. Cilia-associated respiratory bacillus in wild rats in central Iowa. J Wildl Dis. 1993;29(1):123–6. doi: 10.7589/0090-3558-29.1.123 . 8445771

[25] Kakrada MK, Lumsden JS, Lee EA, Collett MG. Cilia-associated respiratory bacillus infection in rats in New Zealand. N Z Vet J. 2002;50(2):81–2. doi: 10.1080/00480169.2002.36255 . 16032215

[26] Jacoby RO, Lindsey JR. Health care for research animals is essential and affordable. FASEB J. 1997;11(8):609–14. doi: 10.1096/fasebj.11.8.9240962 . 9240962

[27] Ramos-Vara JA, Franklin C, Miller MA. Bronchitis and bronchiolitis in a cat with cilia-associated respiratory bacillus-like organisms. Vet Pathol. 2002;39(4):501–4. doi: 10.1354/vp.39-4-501 . 12126155

[28] Barthold SWG, Stephen M., Percy DH. Pathology of laboratory rodents and rabbits. 4 ed. Iowa, USA: John Wiley and Sons, Inc.; 2016.

[29] Wilson R, Cole PJ. The effect of bacterial products on ciliary function. Am Rev Respir Dis. 1988;138(6 Pt 2):S49–53. doi: 10.1164/ajrccm/138.6_Pt_2.S49 . 3202522

[30] Kendall LV, Riley LK, Hook RR, Besch-Williford CL, Franklin CL. Antibody and cytokine responses to the cilium-associated respiratory bacillus in BALB/c and C57BL/6 mice. Infect Immun. 2000;68(9):4961–7. doi: 10.1128/iai.68.9.4961-4967.2000 ; PubMed Central PMCID: PMC101710. 10948111 PMC101710

[31] Matsushita S, Kashima M, Joshima H. Serodiagnosis of cilia-associated respiratory bacillus infection by the indirect immunofluorescence assay technique. Lab Anim. 1987;21(4):356–9. doi: 10.1258/002367787781363327 . 3320514

[32] Cundiff DD, Besch-Williford C, Hook RR, Franklin CL, Riley LK. Detection of cilia-associated respiratory bacillus by PCR. J Clin Microbiol. 1994;32(8):1930–4. doi: 10.1128/JCM.32.8.1930-1934.1994 ; PubMed Central PMCID: PMC263905. 7989545 PMC263905

[33] Trzil JE. Feline Asthma: Diagnostic and Treatment Update. Vet Clin North Am Small Anim Pract. 2019. Epub 2019/12/04. doi: 10.1016/j.cvsm.2019.10.002 . 31812220

[34] Padrid PH. Chronic bronchitis and asthma in cats. Chicago, Illinois: Current veterinary therapy XIV. Philadelphia: WB Saunders.; 2009. p. 650–8.

[35] Lee EA, Johnson LR, Johnson EG, Vernau W. Clinical features and radiographic findings in cats with eosinophilic, neutrophilic, and mixed airway inflammation (2011–2018). J Vet Intern Med. 2020;34(3):1291–9. Epub 2020/04/27. doi: 10.1111/jvim.15772 ; PubMed Central PMCID: PMC7255660. 32338397 PMC7255660

[36] Grotheer M, Hirschberger J, Hartmann K, Castelletti N, Schulz B. Comparison of signalment, clinical, laboratory and radiographic parameters in cats with feline asthma and chronic bronchitis. J Feline Med Surg. 2020;22(7):649–55. Epub 2019/09/04. doi: 10.1177/1098612X19872428 . 31483195 PMC10814432

[37] Reinero CR, Masseau I, Grobman M, Vientos-Plotts A, Williams K. Perspectives in veterinary medicine: Description and classification of bronchiolar disorders in cats. J Vet Intern Med. 2019;33(3):1201–21. Epub 2019/04/13. doi: 10.1111/jvim.15473 ; PubMed Central PMCID: PMC6524100. 30982233 PMC6524100

[38] van Eeden ME, Vientós-Plotts AI, Cohn LA, Reinero CR. Serum allergen-specific IgE reactivity: is there an association with clinical severity and airway eosinophilia in asthmatic cats? J Feline Med Surg. 2020;22(12):1129–36. Epub 2020/03/13. doi: 10.1177/1098612X20907178 . 32167403 PMC10814376

[39] Faner R, Sibila O, Agustí A, Bernasconi E, Chalmers JD, Huffnagle GB, et al. The microbiome in respiratory medicine: current challenges and future perspectives. Eur Respir J. 2017;49(4). Epub 2017/04/12. doi: 10.1183/13993003.02086-2016 . 28404649

[40] Mathieu E, Escribano-Vazquez U, Descamps D, Cherbuy C, Langella P, Riffault S, et al. Paradigms of Lung Microbiota Functions in Health and Disease, Particularly, in Asthma. Front Physiol. 2018;9:1168. Epub 2018/08/21. doi: 10.3389/fphys.2018.01168 ; PubMed Central PMCID: PMC6110890. 30246806 PMC6110890

[41] Caverly LJ, Huang YJ, Sze MA. Past, Present, and Future Research on the Lung Microbiome in Inflammatory Airway Disease. Chest. 2019;156(2):376–82. Epub 2019/05/30. doi: 10.1016/j.chest.2019.05.011 . 31154042 PMC6945648

[42] Vientós-Plotts AI, Ericsson AC, Rindt H, Grobman ME, Graham A, Bishop K, et al. Dynamic changes of the respiratory microbiota and its relationship to fecal and blood microbiota in healthy young cats. PLoS One. 2017;12(3):e0173818. Epub 2017/03/09. doi: 10.1371/journal.pone.0173818 ; PubMed Central PMCID: PMC5344508. 28278278 PMC5344508

[43] Dorn ES, Tress B, Suchodolski JS, Nisar T, Ravindran P, Weber K, et al. Bacterial microbiome in the nose of healthy cats and in cats with nasal disease. PLoS One. 2017;12(6):e0180299. Epub 2017/06/29. doi: 10.1371/journal.pone.0180299 ; PubMed Central PMCID: PMC5491177. 28662139 PMC5491177

[44] Fastrès A, Taminiau B, Vangrinsven E, Tutunaru AC, Moyse E, Farnir F, et al. Effect of an antimicrobial drug on lung microbiota in healthy dogs. Heliyon. 2019;5(11):e02802. Epub 2019/11/14. doi: 10.1016/j.heliyon.2019.e02802 ; PubMed Central PMCID: PMC6895694. 31844730 PMC6895694

[45] Naceradska M, Pekova S. First identification of Filobacterium rodentium as a causative agent of bronchial disease in two cats (Abstract). Journal of Feline Medicine and Surgery. 2019;(21):843–52. 10.1177/1098612X19870390.

[46] Singletary GE, Rush JE, Fox PR, Stepien RL, Oyama MA. Effect of NT-pro-BNP assay on accuracy and confidence of general practitioners in diagnosing heart failure or respiratory disease in cats with respiratory signs. J Vet Intern Med. 2012;26(3):542–6. Epub 2012/03/28. doi: 10.1111/j.1939-1676.2012.00916.x . 22458368

[47] Andreasen CB. Bronchoalveolar lavage. Vet Clin North Am Small Anim Pract. 2003;33(1):69–88. doi: 10.1016/s0195-5616(02)00056-6 . 12512377

[48] Pekova S, Vydra J, Kabickova H, Frankova S, Haugvicova R, Mazal O, et al. Candidatus *Neoehrlichia mikurensis* infection identified in 2 hematooncologic patients: benefit of molecular techniques for rare pathogen detection. Diagn Microbiol Infect Dis. 2011;69(3):266–70. doi: 10.1016/j.diagmicrobio.2010.10.004 . 21353949

[49] Langan G, Lohmiller J, Swing S, Wardrip C. Respiratory diseases of rodents and rabbits. Veterinary Clinics: Small Animal Practice; 2000. p. 1309–35. doi: 10.1016/s0195-5616(00)06009-5 11221984 PMC7134472

[50] Matsushita S, Suzuki E. Prevention and treatment of cilia-associated respiratory bacillus in mice by use of antibiotics. Lab Anim Sci. 1995;45(5):503–7. . 8569147

[51] Habermann RT, Williams FP, McPherson CW, Every RR. The effect of orally administered sulfamerazine and chlortetracycline on chronic respiratory disease in rats. Lab Anim Care. 1963;13:28–40. . 13951654

[52] Brook I, Wexler HM, Goldstein EJ. Antianaerobic antimicrobials: spectrum and susceptibility testing. Clin Microbiol Rev. 2013;26(3):526–46. doi: 10.1128/CMR.00086-12 ; PubMed Central PMCID: PMC3719496. 23824372 PMC3719496

[53] Ahmed B, Cox MJ, Cuthbertson L, James PL, Cookson WOC, Davies JC, et al. Comparison of the upper and lower airway microbiota in children with chronic lung diseases. PLoS One. 2018;13(8):e0201156. Epub 2018/08/02. doi: 10.1371/journal.pone.0201156 ; PubMed Central PMCID: PMC6071972. 30071000 PMC6071972

[54] Aliouat-Denis CM, Chabé M, Demanche C, Aliouat eM, Viscogliosi E, Guillot J, et al. *Pneumocystis* species, co-evolution and pathogenic power. Infect Genet Evol. 2008;8(5):708–26. Epub 2008/05/09. doi: 10.1016/j.meegid.2008.05.001 . 18565802

[55] Boltin D, Niv Y, Schütte K, Schulz C. Review: *Helicobacter pylori* and non-malignant upper gastrointestinal diseases. Helicobacter. 2019;24 Suppl 1:e12637. doi: 10.1111/hel.12637 . 31486237

[56] Rozanski EA, Hoffman AM. Pulmonary function testing in small animals. Clin Tech Small Anim Pract. 1999;14(4):237–41. doi: 10.1016/S1096-2867(99)80017-6 . 10652842

[57] Hawkins EC, DeNicola DB, Kuehn NF. Bronchoalveolar lavage in the evaluation of pulmonary disease in the dog and cat. State of the art. J Vet Intern Med. 1990;4(5):267–74. doi: 10.1111/j.1939-1676.1990.tb03120.x . 2262929

[58] Bannasch MJ, Foley JE. Epidemiologic evaluation of multiple respiratory pathogens in cats in animal shelters. J Feline Med Surg. 2005;7(2):109–19. doi: 10.1016/j.jfms.2004.07.004 . 15771947 PMC10822251

[59] Foster SF, Martin P, Braddock JA, Malik R. A retrospective analysis of feline bronchoalveolar lavage cytology and microbiology (1995–2000). J Feline Med Surg. 2004;6(3):189–98. doi: 10.1016/j.jfms.2003.12.001 . 15135356 PMC10822342

[60] Foster SF, Barrs VR, Martin P, Malik R. Pneumonia associated with Mycoplasma spp in three cats. Aust Vet J. 1998;76(7):460–4. doi: 10.1111/j.1751-0813.1998.tb10178.x . 9700396

[61] Foster SF, Martin P. Lower respiratory tract infections in cats: reaching beyond empirical therapy. J Feline Med Surg. 2011;13(5):313–32. doi: 10.1016/j.jfms.2011.03.009 ; PubMed Central PMCID: PMC7129729. 21515220 PMC7129729

[62] N. A. Use of nebulisers in small animal practice. The Veterinary Nurse. 2015;6(8):488–93.

[63] Van N. I. Bronchial disease in the dog and cat. UK Vet Companion Animal. 2006;11(2):45.

[64] Radford AD, Addie D, Belák S, Boucraut-Baralon C, Egberink H, Frymus T, et al. Feline calicivirus infection. ABCD guidelines on prevention and management. J Feline Med Surg. 2009;11(7):556–64. doi: 10.1016/j.jfms.2009.05.004 . 19481035 PMC11132273

[65] Thiry E, Addie D, Belák S, Boucraut-Baralon C, Egberink H, Frymus T, et al. Feline herpesvirus infection. ABCD guidelines on prevention and management. J Feline Med Surg. 2009;11(7):547–55. doi: 10.1016/j.jfms.2009.05.003 . 19481034 PMC7129359

[66] Egberink H, Addie D, Belák S, Boucraut-Baralon C, Frymus T, Gruffydd-Jones T, et al. Bordetella bronchiseptica infection in cats. ABCD guidelines on prevention and management. J Feline Med Surg. 2009;11(7):610–4. doi: 10.1016/j.jfms.2009.05.010 . 19481041 PMC11132281

[67] Lappin MR, Blondeau J, Boothe D, Breitschwerdt EB, Guardabassi L, Lloyd DH, et al. Antimicrobial use Guidelines for Treatment of Respiratory Tract Disease in Dogs and Cats: Antimicrobial Guidelines Working Group of the International Society for Companion Animal Infectious Diseases. J Vet Intern Med. 2017;31(2):279–94. Epub 2017/02/10. doi: 10.1111/jvim.14627 ; PubMed Central PMCID: PMC5354050. 28185306 PMC5354050

[68] Neiger R, Simpson KW. Helicobacter infection in dogs and cats: facts and fiction. J Vet Intern Med. 2000;14(2):125–33. doi: 10.1892/0891-6640(2000)014<0125:iidacf>2.3.co;2 . 10772482

