# Supplementary figures and images for: A novel Filobacterium sp can cause chronic bronchitis in cats
Source: PLoS One. 2021 Jun 9;16(6):e0251968. doi: 10.1371/journal.pone.0251968 (PMC8189514; doi:10.1371/journal.pone.0251968)

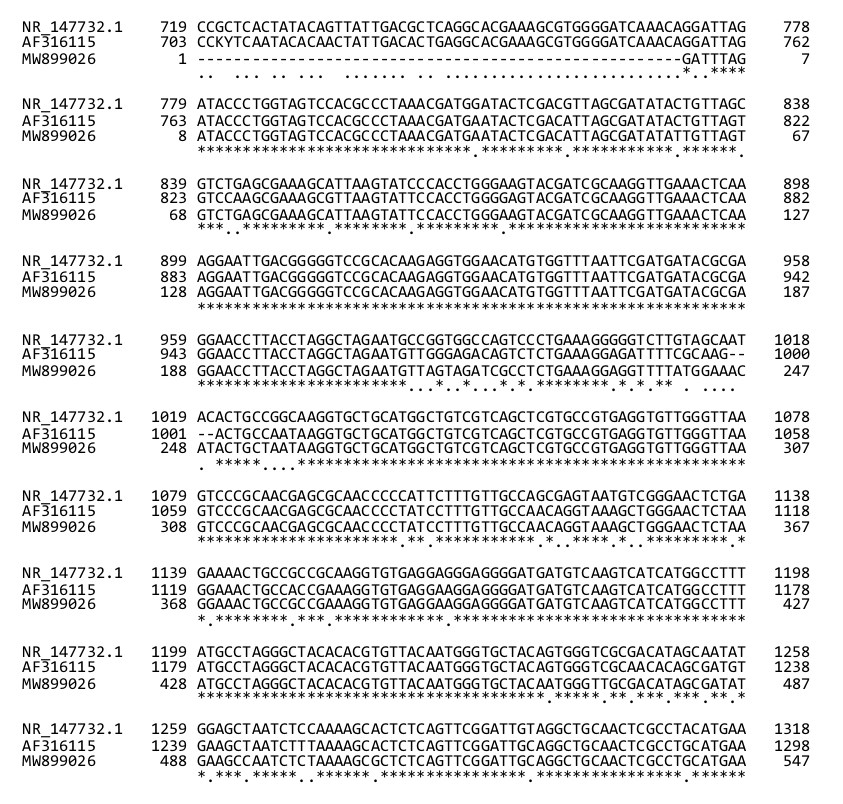

Supplement: S1 Fig — (TIF) [file pone.0251968.s001.tif]
