# Supplementary material for: A novel Filobacterium sp can cause chronic bronchitis in cats
Source: PLoS One. 2021 Jun 9;16(6):e0251968. doi: 10.1371/journal.pone.0251968 (PMC8189514; doi:10.1371/journal.pone.0251968)
Supplement: S1 Table — (DOCX) [file pone.0251968.s002.docx]

S1 Table. Biochemistry results of 3 Czech BAL examined cats

| **Biochemistry** | **Cat 1** | **Cat 2** | **Cat 3** | **Reference interval** |
| --- | --- | --- | --- | --- |
| Urea | 8.4 | 10.1 | 8.7 | 5.7 - 13.5 mmol/l |
| Creatinine | 124 | 90 | 146 | < 168 µmol/l |
| Sodium | 158 | 158 |  | 147 - 159 mmol/l |
| Chloride | 119 | 120 |  | 109 - 129 mmol/l |
| Potassium | 3.8 | 3.7 |  | 3.3 - 5.8 mmol/l |
| Inorganic Phosphate | 1.8 | 1.2 |  | 0.8 - 2.2 mmol/l |
| Bilirubin | 6 | 2.7 |  | < 6.8 µmol/l |
| ALT | 53 | 49 | 74 | < 175 U/l |
| ALP | 39 | 12 | 68 | < 73 U/l |
| GGT | 1 | < 1 |  | < 5 U/l |
| AST | 50 | 21 | 33 | < 71 U/l |
| Total protein | 82 | 64 | 80 | 59 - 87 g/l |
| Albumin | 34 | 37 | 29 | 27 - 44 g/l |
| Globulin | 47 | 27 | 51 | 29 - 54 g/l |
| Glucose | 6.48 | 7.7 | 6.21 | 3.5 - 7.8 mmol/l |
| Cholesterol | 3.39 | 4.0 |  | < 8.5 mmol/l |
| Calcium | 2.81 | 2.5 |  | 2.2 - 2.9 mmol/l |
