# Supplementary material for: A novel Filobacterium sp can cause chronic bronchitis in cats
Source: PLoS One. 2021 Jun 9;16(6):e0251968. doi: 10.1371/journal.pone.0251968 (PMC8189514; doi:10.1371/journal.pone.0251968)
Supplement: S2 Table — (DOCX) [file pone.0251968.s003.docx]

S2 Table. Haematology results of 3 Czech BAL examined cats

| **Haematology** | **Cat 1** | **Cat 2** | **Cat 3** | **Reference interval** |
| --- | --- | --- | --- | --- |
| RBC | 9.18 | 9.0 | 8.65 | 6.51-12.2 T/l |
| HCT | 39.7 | 42.7 | 41.3 | 30.3-52.3 % |
| MCV | 43.2 | 52.6 | 47.7 | 35.9-53.1 fl |
| PLT | 456 | 256 | 272 | 151-600 G/l |
| WBC | 11.72 | 4.9 | 11.91 | 2.87-17.02 G/l |
| HGB | 14.3 | 14.8 | 13.5 | 9.8-16.2 g/dl |
| MCH | 15.6 | 16.4 | 15.6 | 11.8-17.3 pg |
| MCHC | 36.0 | 31.2 | 32.7 | 28.1-35.8 g/dl |
| Neutrophils (segmented) | 42.9 | 63 | 75.5 | 50-75% |
| Neutrophils bands | 0 | 0 | 0 |  |
| Lymphocytes | 35.0 | 24 | 20.3 | 15-50% |
| Eosinophils | 18.3 | 10 | 1.3 | 0-6% |
| Monocytes | 2.1 | 3 | 1.2 | 0-4% |
| Basophils | 1.7 | 0 | 1.7 | 0-1% |
